# Massive Hemoperitoneum Caused by Rupture of a Superficial Vessel on a Large Pedunculated Uterine Leiomyoma: A Case Report

**DOI:** 10.7759/cureus.98915

**Published:** 2025-12-10

**Authors:** Riho Seki, Takeshi Fukuda, Reiko Tasaka, Takuma Wada, Toshiyuki Sumi

**Affiliations:** 1 Department of Obstetrics and Gynecology, Osaka Metropolitan University Graduate School of Medicine, Osaka, JPN

**Keywords:** abdominal pain, hemoperitoneum, hemorrhagic shock, rupture of a superficial vessel, uterine leiomyoma

## Abstract

Spontaneous hemoperitoneum caused by a uterine leiomyoma is an exceptionally rare but potentially life-threatening condition. We report the case of a 40-year-old woman who presented with sudden-onset severe abdominal pain and hemorrhagic shock. Focused sonography revealed massive hemoperitoneum, and contrast-enhanced computed tomography demonstrated intra-abdominal bleeding associated with a large uterine mass. Emergency laparotomy revealed a large pedunculated leiomyoma with rupture of a superficial vessel on its surface, without evidence of torsion. Approximately 2,250 mL of blood was lost, and myomectomy was performed because the fibroid could be rapidly and safely excised by transecting the stalk, and the patient wished to preserve fertility. The postoperative course was uneventful, and histopathology confirmed a benign leiomyoma. Rupture of superficial vessels overlying uterine fibroids is extremely uncommon but should be considered in women presenting with hemoperitoneum of unknown origin, especially those with large leiomyomas. Early recognition and prompt surgical intervention are essential to prevent severe morbidity or mortality. This case underscores the importance of including the vascular rupture of a leiomyoma in the differential diagnosis of an acute abdomen.

## Introduction

Spontaneous hemoperitoneum in women is a rare but potentially life-threatening condition, with no established incidence due to its occurrence being reported almost exclusively through isolated case reports. The etiologies vary depending on age and reproductive status. In reproductive-aged women, the most common causes include ruptured ectopic pregnancy, hemorrhagic corpus luteum, and ruptured ovarian cyst [1]. In contrast, in nonpregnant women without ovarian pathology, spontaneous intraperitoneal hemorrhage of uterine origin is exceedingly uncommon.

Among gynecologic causes, uterine leiomyoma is one of the most frequent benign tumors, occurring in up to 70% of women by the age of 50 [2]. While most leiomyomas are asymptomatic or cause chronic pelvic symptoms such as menorrhagia, dysmenorrhea, pelvic pain, and infertility, which are well known and fully covered by the literature, acute presentations are rare [3]. Spontaneous hemoperitoneum due to rupture of a superficial vessel overlying a subserosal or pedunculated leiomyoma represents an exceptionally rare but potentially fatal complication [4].

Given its rarity and diagnostic challenge, clinicians should be aware of this entity as a possible cause of spontaneous hemoperitoneum in women of reproductive age. Here, we report a case of massive hemoperitoneum caused by rupture of a superficial vessel overlying a large pedunculated uterine leiomyoma, successfully managed with emergency surgery.

## Case presentation

A 40-year-old woman presented to emergency medical services (EMS) with sudden-onset, severe right lower abdominal pain. Her blood pressure and pulse rate at the time of EMS contact were 76/44 mmHg and 100 bpm, and she was urgently transported to the emergency department. Her obstetric history was gravida 3, para 0, and she had no significant medical history. Her last menstrual period had begun one day prior to presentation.

**Figure 1:**
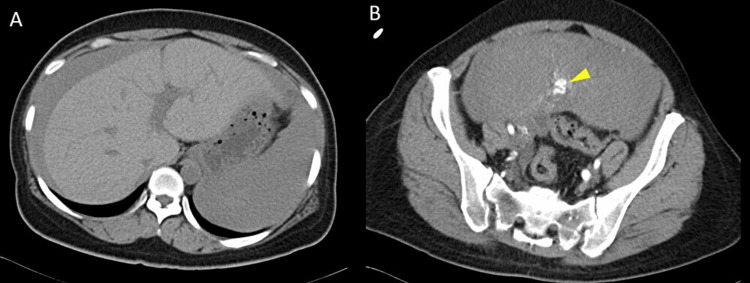
Contrast-enhanced computed tomography (CT). A: CT demonstrating a large volume of hemoperitoneum surrounding the liver; B: contrast-enhanced CT demonstrating a large uterine mass, presumed to be a leiomyoma, with prominent contrast-enhanced vessels visible along its surface, as indicated by the arrowhead.

On arrival, the patient appeared in hemorrhagic shock, with a blood pressure of 77/49 mmHg, a heart rate of 100 beats per minute, and oxygen saturation of 98% on room air. Laboratory evaluation revealed a hemoglobin concentration of 10.3 g/dL, a red blood cell count of 348 × 104/µL, and a hematocrit of 29.8%. Focused assessment with sonography for trauma demonstrated massive hemoperitoneum and a large pelvic mass consistent with a uterine mass (no images were available because the scan was performed using a portable ultrasound device). Contrast-enhanced computed tomography confirmed extensive intra-abdominal bleeding, likely originating from the uterine fibroid (Figure 1).

**Figure 2:**
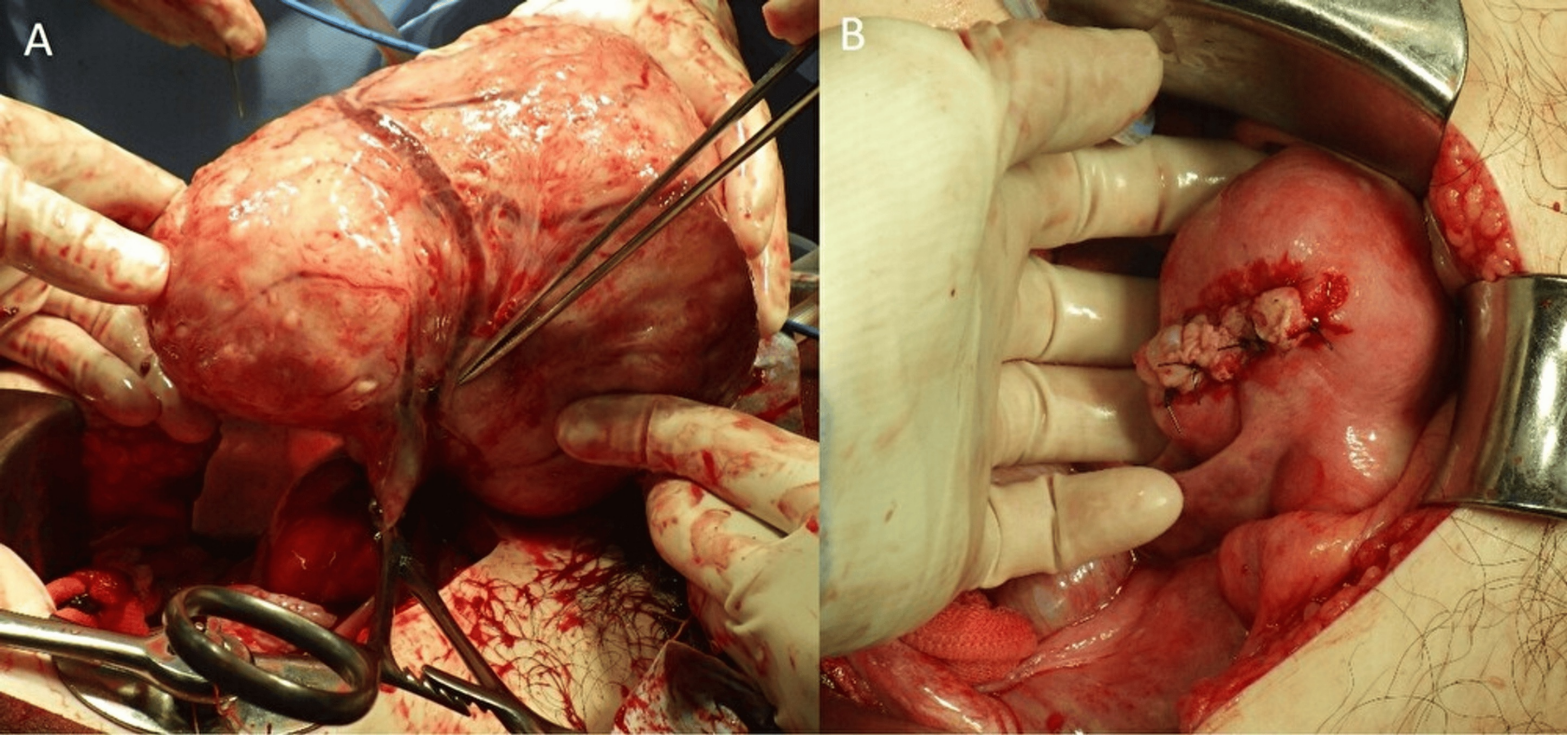
Intraoperative findings of the pedunculated uterine leiomyoma and surgical management. A: intraoperative picture showing a pedunculated uterine leiomyoma delivered outside the abdominal cavity, the stalk is clamped with forceps, the surgeon is pointing to the ruptured superficial vein on the tumor surface, and the hemorrhage is controlled due to clamping of the stalk; B: intraoperative picture after removal of the pedunculated leiomyoma and closure of the myometrial defect.

An emergency laparotomy was performed. Through an infraumbilical midline incision, approximately 1,350 mL of blood was evacuated from the peritoneal cavity. A large pedunculated leiomyoma, approximately the size of a newborn’s head, was identified on the fundus of the uterus. The superficial vessel was disrupted, and active bleeding was noted at the same site. No torsion of the tumor was present. Both ovaries appeared normal. Because the patient desired preservation of fertility, a myomectomy was performed (Figure 2).

The total estimated blood loss was 2,250 mL, and the patient received eight units of packed red blood cells and two units of fresh frozen plasma intraoperatively. Her postoperative recovery was uneventful, and her laboratory data improved to a hemoglobin concentration of 11.0 g/dL, a red blood cell count of 369 × 104/µL, and a hematocrit of 30.2%. She was discharged on postoperative day 6.

Histopathological examination confirmed a benign leiomyoma without evidence of malignancy. At her four-week postoperative follow-up, she reported no symptoms, and no abnormal findings were observed.

## Discussion

Spontaneous hemoperitoneum caused by a uterine leiomyoma is an extremely rare but potentially life-threatening event. Although uterine leiomyomas are highly prevalent, with some studies reporting a lifetime incidence as high as 70% [5]; vascular rupture leading to massive intra-abdominal bleeding occurs only in exceptional cases. Approximately 125 cases of hemoperitoneum due to leiomyoma have been documented in the literature [6], with only about 30 attributed to rupture of a superficial vessel on the surface of the leiomyoma [6]. The rarity of this condition is particularly striking considering the high prevalence of leiomyomas in reproductive-aged women.

Pathophysiological mechanisms

Several mechanisms have been proposed to explain spontaneous vascular rupture from leiomyomas. Two classical theories focus on venous rupture [7]. The first suggests that leiomyomas larger than 10 cm exert pressure on overlying veins, causing thinning and eventual tearing. The second relates the tear in the blood vessel to uterine contractions during menstruation that distend the superficial veins to the breaking point [7]. Notably, many reported cases, including ours, occurred during menstruation, supporting this theory.

Additional contributing factors include increased intra-abdominal pressure due to pregnancy, constipation, strenuous exercise, defecation, abdominal massage, coughing, sexual intercourse, and weight-bearing activities [3]. Venous congestion may also be promoted by nulliparity, age 30-49, hormonal therapy, or large size of leiomyoma (>10 cm) [3].

In the present case, the absence of torsion and the presence of disrupted superficial nutrient vessels on the surface of a large pedunculated leiomyoma suggest that mechanical stretching and congestion of the overlying venous structures may have contributed to the rupture.

While most bleeding is venous in origin, accounting for up to 76.3% of cases [8], arterial rupture, although far less common (11.3%) [8], has been documented and is associated with severe hemodynamic compromise [4].

Vascular anatomy of leiomyomas

The vascular architecture of leiomyomas further explains their vulnerability to rupture. Leiomyomas are supplied by distorted, tortuous, and often enlarged branches of the uterine artery [9,10]. The parenchyma of the leiomyoma itself is relatively hypovascular, with small centripetal vessels supplying the internal bulk, whereas the peripheral aspect is nourished by larger arteries than those supplying the normal myometrium [9,10]. Venous drainage occurs through dilated veins traversing the surface of the leiomyoma before entering the peripheral myometrial venous plexus [10]. These vascular abnormalities produce prominent, fragile superficial vessels, especially in leiomyomas larger than 10 cm, making them prone to rupture [9,10]. As leiomyomas enlarge, they may split feeding vessels between uterine masses, further precipitating bleeding [7].

In most cases, intra-abdominal hemorrhage arises from trauma, torsion, or degeneration of a uterine leiomyoma [10]; however, spontaneous rupture without torsion, such as in our patient, is far more unusual.

Clinical presentation and diagnostic challenges

Patients typically present with sudden abdominal pain, hypovolemic shock, and no clear preoperative diagnosis [8]. In women of reproductive age, the most common diagnosis for hemoperitoneum is ruptured ectopic pregnancy [7]. Other differential diagnoses include hemorrhagic corpus luteum, ruptured ovarian cyst, ovarian torsion, and adnexal tumors [7], as well as non-gynecologic causes such as hepatic, splenic, or vascular rupture, or even rupture of an aortic aneurysm [1,11].

Imaging findings often reveal hemoperitoneum and a pelvic mass, but identifying the precise bleeding source preoperatively remains difficult. Thus, exploratory surgery, laparotomy, or laparoscopy, remains the cornerstone of diagnosis and management.

Outcomes and management

Prompt resuscitation and emergency surgery are crucial, as delays may result in significant morbidity or mortality; the reported mortality rate from leiomyoma-related intra-abdominal hemorrhage is approximately 3.2% [8]. Historically, hysterectomy was commonly performed, particularly in women who had completed childbearing. In our case, myomectomy was chosen because the leiomyoma was pedunculated and could be rapidly and safely excised by transecting the stalk, and the patient also wished to preserve fertility.

Clinical implications

Uterine leiomyoma is the most common benign uterine tumor, and its usual complications, such as menorrhagia, dysmenorrhea, pelvic pain, urinary or bowel symptoms, symptomatic anemia, and infertility, are well known and extensively covered in the literature [3,12]. In contrast, acute complications such as torsion of a pedunculated leiomyoma, degeneration, and intra-abdominal hemorrhage are rare but may be catastrophic [1].

Although uncommon, bleeding from fibroid vessels should be considered in women with large leiomyomas who present with unexplained hemoperitoneum, particularly during menstruation. Awareness of this entity is essential for timely diagnosis, rapid surgical intervention, and reduction of morbidity associated with catastrophic intra-abdominal bleeding.

## Conclusions

Spontaneous hemoperitoneum resulting from rupture of a superficial vessel overlying a uterine leiomyoma is an exceedingly rare but potentially life-threatening condition. Because the clinical presentation is often nonspecific and preoperative diagnosis is challenging, clinicians must maintain a high index of suspicion, particularly in reproductive-aged women who present with acute abdominal pain, hemodynamic instability, and known or suspected fibroids. Prompt recognition, rapid resuscitation, and timely surgical intervention are essential to prevent serious morbidity or mortality.

Given that large leiomyomas, particularly those measuring 10 cm or more, are associated with stretching and fragility of superficial vessels, clinicians should consider the risk of intraperitoneal hemorrhage when counseling such patients. Furthermore, awareness of this rare complication is important not only for gynecologists but also for physicians in other specialties, especially emergency medicine, as these patients may initially present outside gynecologic care. This case underscores the importance of including vascular rupture of a leiomyoma in the differential diagnosis of unexplained hemoperitoneum and demonstrates that, in selected patients, fertility-preserving surgery such as myomectomy can be successfully performed.
